# Ovarian Torsion in Patients With Polycystic Ovary Syndrome Without Ovulation Induction or an Adnexal Mass: A Report of Two Cases

**DOI:** 10.7759/cureus.88608

**Published:** 2025-07-23

**Authors:** Abbas Abuelhassan

**Affiliations:** 1 Obstetrics and Gynaecology, Ashford and St. Peter's Hospitals NHS Foundation Trust, Surrey, GBR

**Keywords:** anovulation, diagnostic laparoscopy, ovarian cyst torsion, ovarian detorsion, polycystic ovary syndrome (pcos)

## Abstract

Ovarian torsion is a gynaecological emergency, often linked with adnexal masses or cysts. Nonetheless, emerging reports indicate that torsion may also occur in polycystic ovary syndrome (PCOS), even in the absence of overt structural abnormalities. We present two cases of ovarian torsion in women with PCOS, neither of whom had large cysts, masses, or prior ovulation induction. These cases emphasize the need for heightened clinical suspicion when evaluating lower abdominal pain in PCOS patients. Incorporating imaging findings, surgical interventions, and a review of current literature, we aim to reinforce awareness of this uncommon but critical scenario.

## Introduction

Ovarian torsion, the rotation of the ovary on its ligamentous supports, is a potentially ovary-compromising emergency. It is commonly associated with ovarian masses greater than 5 cm, cysts, or ovulation induction therapy. However, torsion can also develop in polycystic ovaries that exhibit increased size and mobility, even without external triggers [1,2].

Polycystic ovary syndrome (PCOS) is the most common endocrinopathy among women of reproductive age, impacting 5%-10% of premenopausal American women. During the reproductive years, women with PCOS often seek medical attention related to infertility, hirsutism, and acne. About 60% of women with PCOS are obese and insulin resistant [3].

Morphologically, the ovaries are often enlarged, containing numerous small follicles arranged peripherally around increased stromal tissue. These features increase the ovarian volume and weight, which may predispose to mechanical complications like torsion - especially in the absence of anchoring from regular ovulatory cycles.

Patients with PCOS often present with menstrual irregularities, infertility, obesity, and insulin resistance, placing them at risk of delayed diagnosis in emergency scenarios [1]. These structural and morphological changes in PCOS may predispose the ovary to torsion by increasing weight and altering the centre of gravity. The diagnostic challenge is heightened when there are no masses or cysts evident, especially as initial imaging, such as computed tomography (CT), may be inconclusive [4]. We report two such cases and correlate them with recent literature.

## Case presentation

Case 1

A 33-year-old woman, P2 with known PCOS, presented to the Emergency Department with progressively worsening pain in the right iliac fossa (RIF), which had begun several hours prior to arrival. The pain began centrally before localizing to the right side, was rated 10 out of 10 in severity, and was associated with nausea and vomiting. Her menstrual cycles were irregular, with the last proper period noted several months prior. She was not on ovulation-inducing medications or hormonal treatment.

Clinical Examination and Investigations

**Figure 1:**
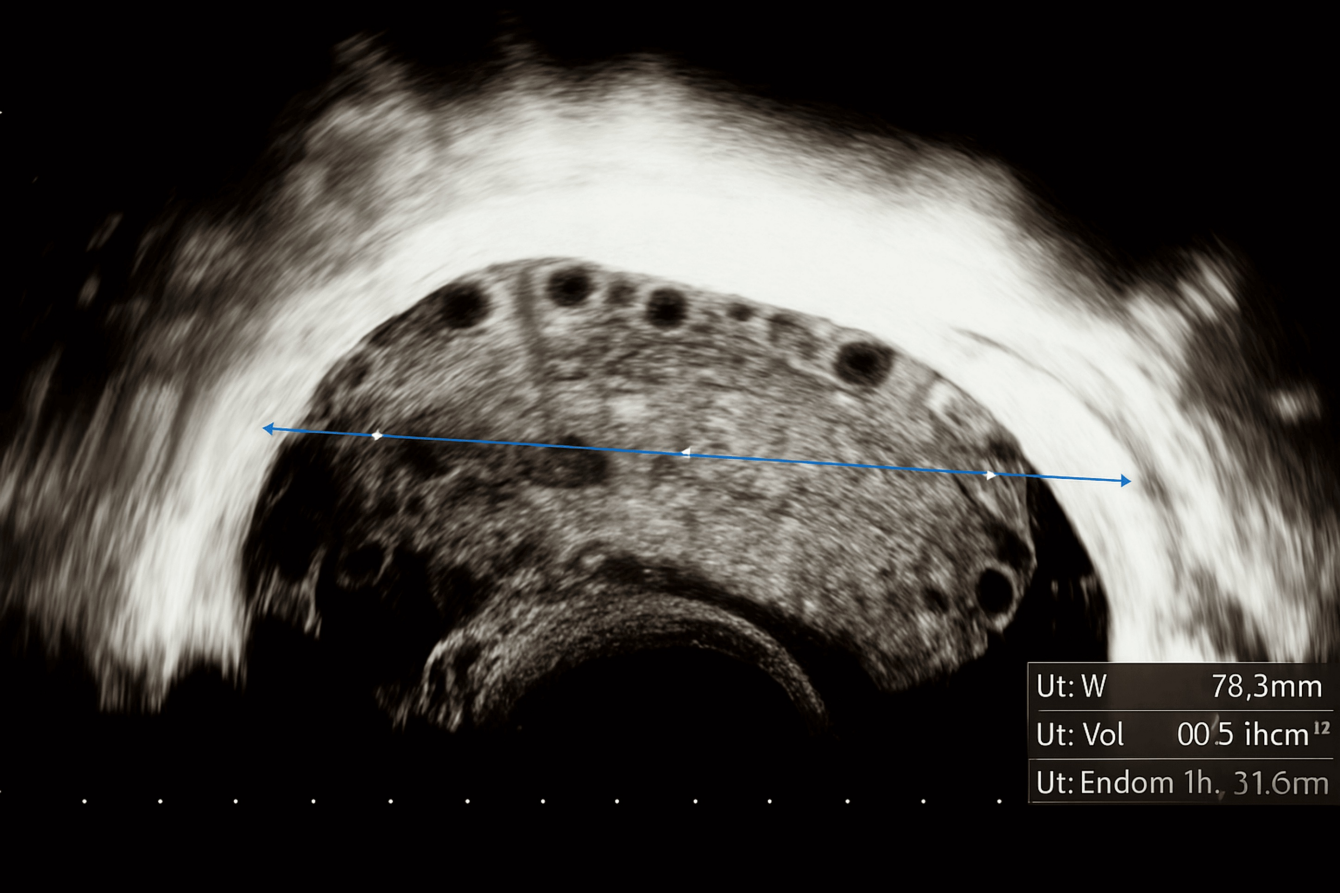
TVUS showing an enlarged right ovary (approximately 7-8 cm, arrow) with peripheral follicles and increased stromal echogenicity. TVUS, Transvaginal ultrasound

The abdomen was soft but tender in the RIF, with voluntary guarding. Speculum exam elicited cervical excitation. Urine pregnancy test was negative. Laboratory findings revealed a normal white cell count and C-reactive protein (CRP) <1. A CT abdomen and pelvis ruled out appendicitis, showing trace pelvic fluid and nonspecific adnexal changes. Transvaginal ultrasound (TVUS) revealed an enlarged right ovary (~7-8 cm), with peripheral follicles and increased stromal echogenicity. Doppler showed reduced vascularity, consistent with torsion (Figure 1).

Surgical Findings and Management

**Figure 2:**
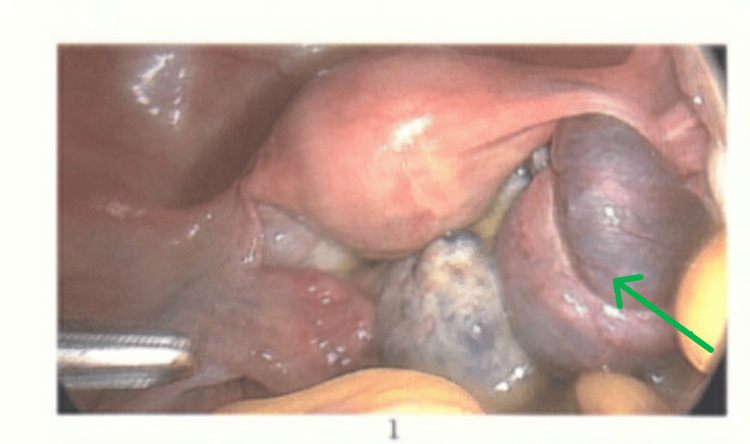
Laparoscopy confirming right ovarian torsion with three twists (arrow).

**Figure 3:**
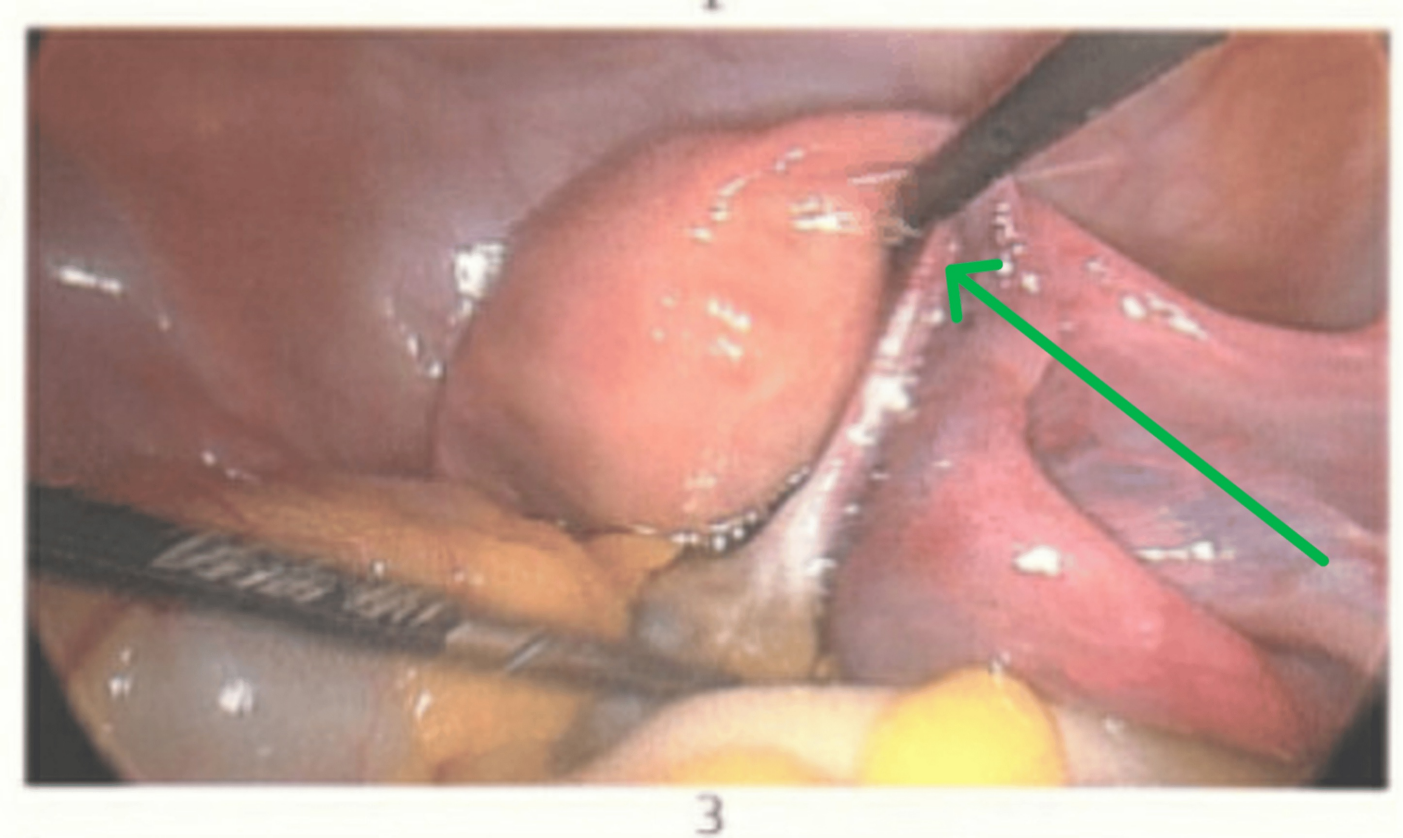
Detorsion is performed as indicated by the arrow.

**Figure 4:**
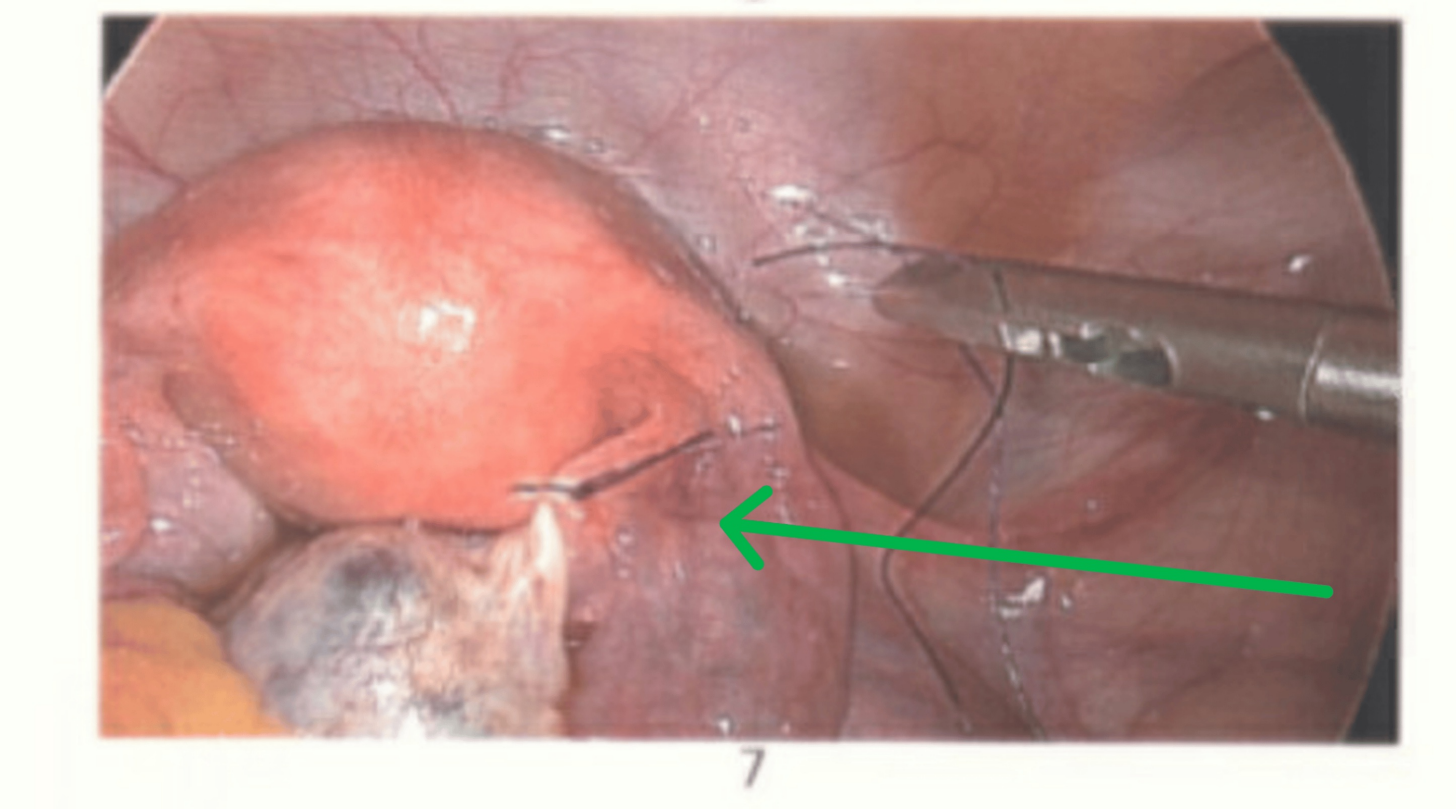
Ovarian ligament is shortened using 2-0 Vicryl suture to reduce the risk of future ovarian torsion (arrow).

Laparoscopy confirmed a right ovarian torsion involving three twists (Figure 2). The ovary appeared polycystic, enlarged, and oedematous. Detorsion was performed (Figure 3), and the ovarian ligament was shortened using 2-0 Vicryl to reduce future torsion risk (Figure 4). The patient recovered uneventfully and was discharged on metformin, with advice regarding hormonal regulation.

Case 2

A 27-year-old woman with a history of PCOS presented with acute right lower quadrant pain for three hours before arrival, gradually worsening throughout the day. The pain was described as sharp, 9 out of 10 in intensity, accompanied by nausea and two episodes of vomiting. Her last regular menstrual cycle occurred over six months prior.

Clinical Examination and Investigations

**Figure 5:**
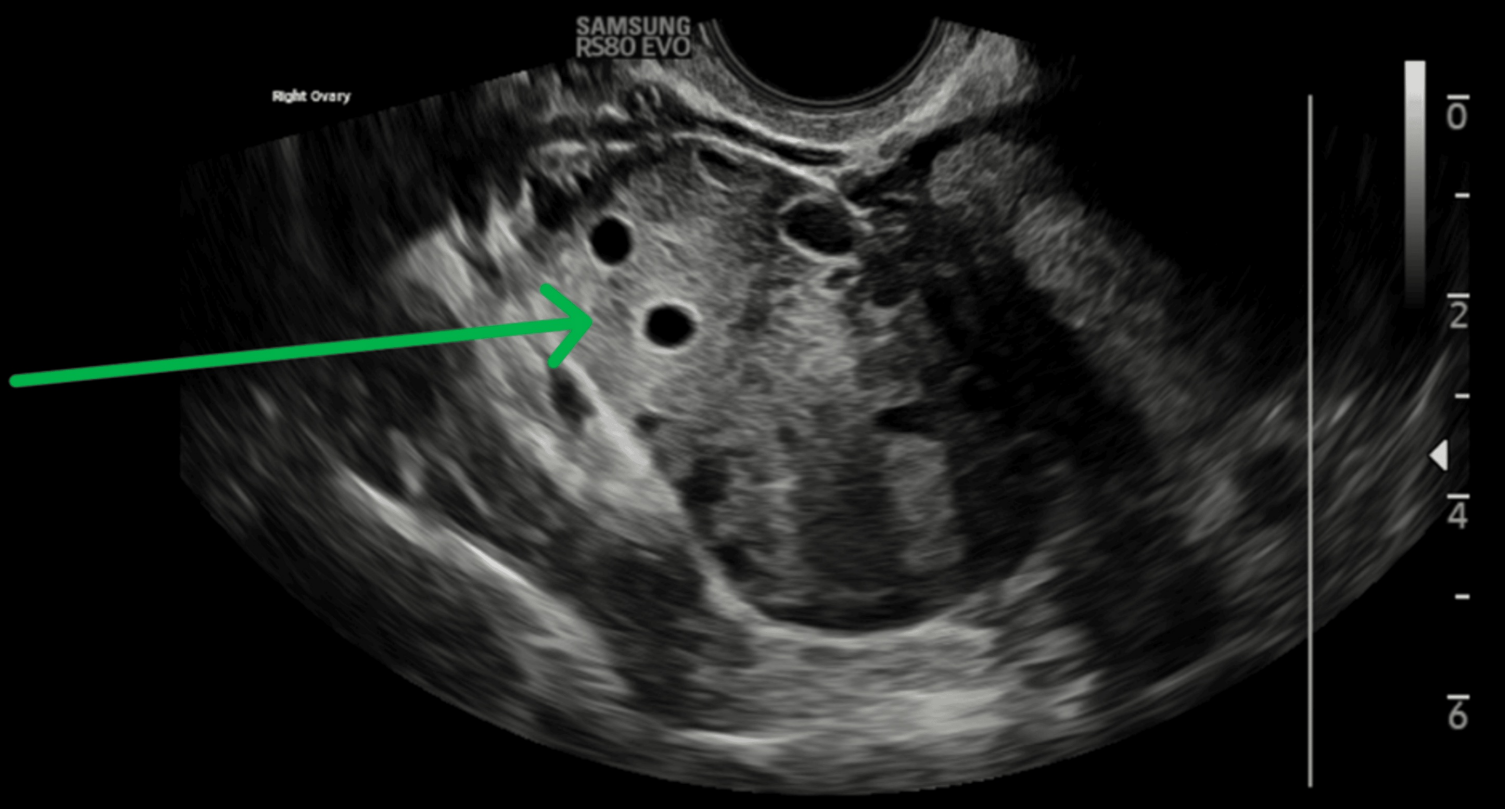
TVUS showing a right ovary measuring 60 mL with classic PCOS morphology: peripherally arranged follicles (arrow). TVUS, Transvaginal ultrasound; PCOS, Polycystic ovary syndrome

Physical examination revealed a soft but tender lower abdomen, with a positive psoas sign. Inflammatory markers were within normal limits. TVUS showed a right ovary measuring 60 mL, with classic PCOS morphology: peripherally arranged follicles and reduced blood flow on Doppler (Figure 5). A small amount of pelvic free fluid was also observed.

Surgical Findings and Management

**Figure 6:**
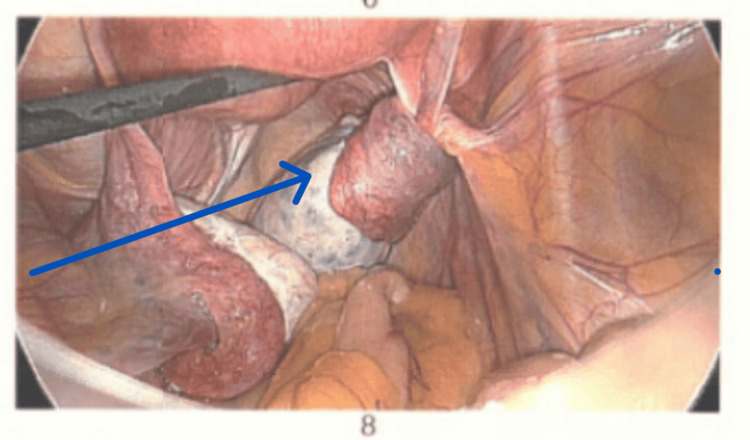
Diagnostic laparoscopy revealing a torted right ovary with no overt cysts or masses (arrow).

**Figure 7:**
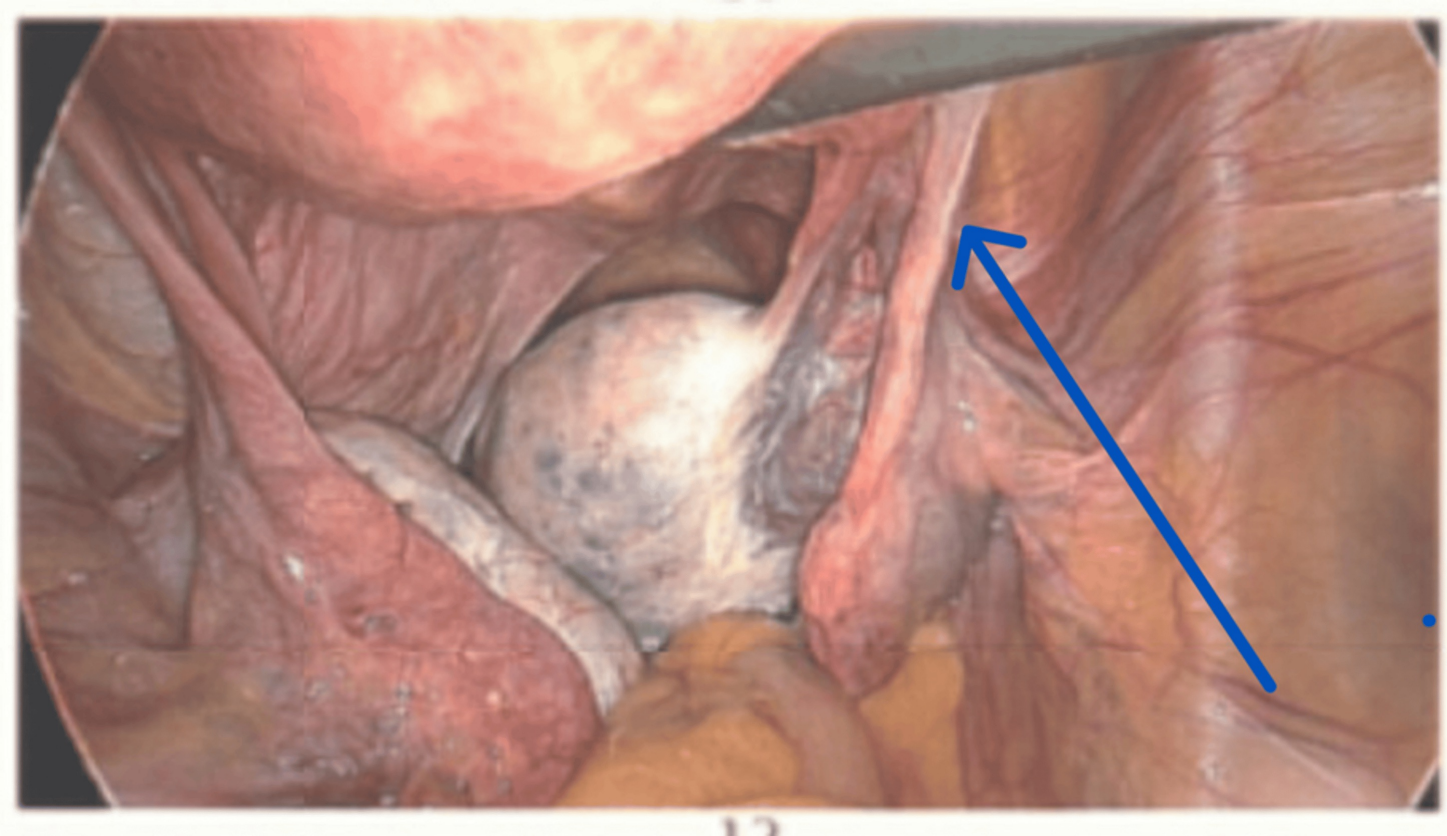
The ovary and tube are viable and successfully detorted (arrow).

Diagnostic laparoscopy revealed a torted right ovary, with no overt cysts or masses (Figure 6). The ovary and tube were viable and were successfully detorted (Figure 7). Minimal pelvic adhesions were present. The patient was discharged the following day, with instructions for follow-up and ongoing PCOS management to be coordinated by her general practitioner.

## Discussion

While adnexal torsion is often linked with large cysts or ovarian masses, ovarian torsion occurs in around 2%-15% of patients who have surgical treatment of adnexal masses [5]. The main risk in ovarian torsion is an ovarian mass [5]. These cases underscore the vulnerability of polycystic ovaries to torsion even in their unprovoked state. PCOS can lead to chronic ovarian enlargement due to antral follicle accumulation and stromal hyperplasia [1]. These anatomical changes, coupled with increased ovarian mobility, heighten torsion risk even without external manipulation or hormonal stimulation.

The diagnostic approach is nuanced. CT imaging may fail to detect torsion, as in Case 1, highlighting the superiority of TVUS with Doppler for assessing ovarian blood flow [4]. Imaging signs, such as increased ovarian volume, peripheral follicle pattern, and reduced vascularity, are suggestive of torsion [2].

Management entails prompt surgical detorsion to salvage ovarian function. In cases of polycystic ovaries, adjunctive measures like ovarian ligament plication may be considered to reduce recurrence risk, as done in Case 1 [6]. Awareness of this atypical presentation can improve timely diagnosis and fertility-preserving outcomes.

## Conclusions

The conclusion rightly asserts that ovarian torsion should be a primary consideration in the differential diagnosis of acute lower abdominal pain in women with PCOS. This is a crucial point, as PCOS itself is characterized by hormonal imbalances and the presence of multiple small cysts on the ovaries, which can predispose women to ovarian torsion. While the absence of overt masses, cysts, or ovulation induction might seem to diminish the likelihood of torsion, this case underscores that these factors are not prerequisites for its occurrence. The inherent anatomical and physiological changes associated with PCOS, such as enlarged ovaries or increased ovarian mobility, can independently elevate the risk of torsion. Therefore, clinicians must maintain a high index of suspicion for ovarian torsion in this patient population, regardless of the typical risk factors often cited in medical literature.
